# Questionnaire data on visual, perceptual, and emotional characteristics of Japanese adults

**DOI:** 10.1016/j.dib.2019.104362

**Published:** 2019-08-05

**Authors:** Shu Imaizumi

**Affiliations:** a Institute for Education and Human Development, Ochanomizu University, 2-1-1 Otsuka, Bunkyo, Tokyo, 112-8610, Japan; b Graduate School of Arts and Sciences, The University of Tokyo, Tokyo, Japan

**Keywords:** Online survey, Visual discomfort, Quality of life, Hypersensitivity, Mood, Disgust

## Abstract

The dataset comprises 344 Japanese adults’ responses to validated psychological scales to assess the following: (1) visual function and its relation to quality of life, (2) susceptibility to visual discomfort, such as aversion to stimuli and eyestrain, (3) hypersensitivity and hyposensitivity across sensory modalities, (4) positive and negative moods, and (5) proneness and sensitivity to disgust. The dataset also includes participants’ gender, age, response times, and dates of participation. The dataset might be used for exploratory analyses and cross-cultural studies.

**Table:** Specifications Table

Subject area	Psychology
More specific subject area	Individual differences, perception, emotion
Type of data	Tables
How data was acquired	Online questionnaire survey
Data format	Raw, filtered, and summarized
Experimental factors	Japanese adults (*n* = 344) completed a battery of psychological scales
Experimental features	Cross-sectional design
Data source location	Crowdsourcing in Japan
Data accessibility	Provided with the article
Related research article	S. Imaizumi, S. Koyama, Y. Tanno, Development of the Japanese version of the Visual Discomfort Scale, PLoS ONE. 13 (2018) e0191094.

**Table:** 

**Value of the data** • Data derived from an online questionnaire survey are useful for exploratory analyses of relationships between psychological constructs on perceptual sensitivities and emotional tendencies, both at the subscale-by-subscale or item-by-item level. • Because the data were derived from validated Japanese versions of psychological scales, they can be used to compare psychometric properties between the Japanese version and versions in other languages. • The data were obtained from a sample of the Japanese adult population and can be used for cross-cultural studies, age comparisons, and patient-control studies.

## 1 Data

The data include responses from Japanese adults to the items of validated psychological scales that assess: (1) visual function and its relation to quality of life, (2) susceptibility to visual discomfort (e.g., aversion to stimuli, eyestrain, reading problems, or headache), (3) hypersensitivity and hyposensitivity across sensory modalities, (4) positive and negative trait moods, and (5) proneness and sensitivity to disgust. Further, the data include gender and age, dates of participation, and response times. The dataset (Supplementary Data) comprises responses from 344 participants and the filtered responses from 326 participants (see the following section). For descriptive purposes, summary statistics of the filtered responses are shown in Table 1.

**Table 1:** Descriptive statistics on a sample of Japanese adults’ responses to the items comprising the visual, perceptual, and emotional psychological scales (*n* = 326).

	Mean (SD)	Median [IQR]	Range	Skewness	Kurtosis	Shapiro-Wilk (*p*)	Cronbach’s *α*
Age (year)	40.2 (10.1)	40 [33, 46]	22–81	0.66	0.60	<.001	N/A
Response time (min.)	18.1 (16.7)	15.0 [11.5, 19.8]	4.4–210.3	7.19	68.55	<.001	N/A
NEI VFQ-25							
General Health	46.8 (20.0)	50.0 [25.0, 50.0]	0–100	−0.03	−0.12	<.001	N/A^∗∗∗^
General Vision	67.9 (16.5)	80.0 [60.0, 80.0]	0–100	−0.91	1.11	<.001	N/A^∗∗∗^
Near Vision	80.8 (18.7)	79.2 [75.0, 100]	0–100	−1.14	1.90	<.001	.881
Distance Vision	82.7 (16.2)	83.3 [75.0, 100]	16.7–100	−1.07	1.23	<.001	.759
Driving^∗^	80.2 (14.6)	87.5 [75.0, 87.5]	37.5–100	−0.71	0.37	<.001	.599
Peripheral Vision^∗∗^	76.2 (19.7)	75.0 [75.0, 100]	0–100	−0.60	0.46	<.001	N/A^∗∗∗^
Color Vision	93.6 (12.3)	100 [100, 100]	50–100	−1.72	2.12	<.001	N/A^∗∗∗^
Ocular Pain	74.4 (19.4)	75.0 [62.5, 87.5]	0–100	−0.73	0.50	<.001	.614
Role Limitations	84.0 (20.7)	87.5 [75.0, 100]	0–100	−1.54	2.78	<.001	.892
Dependency	91.0 (15.2)	100 [83.3, 100]	25–100	−1.92	3.52	<.001	.789
Social Function	89.8 (14.1)	100 [87.5, 100]	25–100	−1.54	2.51	<.001	.772
Mental Health	81.1 (18.5)	87.5 [68.8, 93.8]	12.5–100	−1.17	0.82	<.001	.807
VDS	10.1 (9.4)	7.0 [3.3, 13.8]	0–57	1.57	2.81	<.001	.932
GSQ							
Visual, Hyper	2.31 (2.08)	2 [1, 4]	0–12	1.14	1.70	<.001	.624
Visual, Hypo	2.34 (1.95)	2 [1, 3]	0–12	1.11	1.99	<.001	.561
Auditory, Hyper	6.93 (2.66)	7 [5, 9]	0–12	−0.08	−0.70	<.001	.680
Auditory, Hypo	3.69 (2.22)	4 [2, 5]	0–11	0.59	0.28	<.001	.534
Gustatory, Hyper	3.06 (2.11)	3 [1, 4]	0–11	0.75	0.34	<.001	.572
Gustatory, Hypo	1.44 (1.74)	1 [0, 2]	0–10	1.83	4.10	<.001	.614
Olfactory, Hyper	3.77 (2.71)	3 [2, 6]	0–12	0.73	0.16	<.001	.710
Olfactory, Hypo	1.90 (1.47)	2 [1, 3]	0–7	1.12	1.19	<.001	.477
Tactile, Hyper	2.77 (2.18)	3 [1, 4]	0–12	0.78	0.62	<.001	.336
Tactile, Hypo	2.74 (1.77)	2.5 [1, 4]	0–8	0.54	−0.02	<.001	.439
Vestibular, Hyper	2.91 (2.02)	3 [1, 4]	0–9	0.51	−0.25	<.001	.428
Vestibular, Hypo	1.75 (1.95)	1 [0, 3]	0–11	1.44	2.31	<.001	.647
Proprioceptive, Hyper	2.01 (2.04)	1 [1, 3]	0–12	1.39	2.34	<.001	.592
Proprioceptive, Hypo	2.04 (1.89)	2 [0, 3]	0–10	0.93	0.69	<.001	.569
PANAS							
Positive Affect	30.7 (7.0)	31 [27, 35]	10–50	−0.02	0.25	.107	.837
Negative Affect	32.1 (9.1)	32 [26, 39]	10–57	0.21	−0.21	.078	.900
DPSS-R							
Disgust Propensity	19.5 (6.3)	19 [15, 23]	8–40	0.70	0.60	<.001	.897
Disgust Sensitivity	16.4 (6.5)	15 [11, 20]	8–40	0.97	0.86	<.001	.883

*Note.*^∗^*n* = 194; ^∗∗^*n* = 313 due to “not applicable” responses; ^∗∗∗^these subscales comprise single items. NEI VFQ-25: 25-item National Eye Institute Visual Function Questionnaire; VDS: Visual Discomfort Scale; GSQ: Glasgow Sensory Questionnaire; PANAS: Positive and Negative Affect Schedule; DPSS-R: Disgust Propensity and Sensitivity Scale-Revised; IQR: interquartile range.

## 2 Experimental design, materials, and methods

### 2.1 Survey procedure

Data was obtained from an online, cross-sectional survey conducted in January 2017, in which 344 Japanese adults (173 females; mean age 40.1 years, SD = 10.0, range 22–81) voluntarily participated on Lancers, a crowdsourcing platform available in Japan, using their personal computers. Questionnaire presentation and data collection were performed on the Qualtrics survey website. The survey website first presented the ethical statement and consent form. The participants who gave their informed consent reported their gender and age and completed the battery of five psychological scales (see Measures) presented to them in a pseudo-random order. Last, participants were paid 108 JPY (about 1 USD) as a token of appreciation for their participation.

Probably because of repeated participation, 18 participants had an identical IP address, gender, and age. To ensure data quality, these cases were considered invalid [1]. The filtered data on 326 participants (165 females) are shown in Table 1 and the Supplementary Data. Jamovi version 0.9.6.9 (https://www.jamovi.org) was used to calculate the summary statistics. The ethics committee of the Graduate School of Arts and Sciences, The University of Tokyo, approved this survey.

### 2.2 Measures

The 25-item National Eye Institute Visual Function Questionnaire (NEI VFQ-25) [2], Japanese version [3], is a self-report index comprising 12 subscales (e.g., General Vision, Ocular Pain; Table 1) to assess visual functionality and its relation to quality of life on a daily basis. The items were answered using different Likert-type scales. Subscale scores were standardized to a scale ranging from 0 to 100. A higher score indicates a better visual function and quality of life.

The Visual Discomfort Scale (VDS) [4], Japanese version [5], is a 23-item index with a single factor structure. The VDS assesses frequency of daily experiences of visual discomfort, such as unpleasantness, eye fatigue, and headache induced by visual stimuli (e.g., striped pattern, fluorescent lights). The items are answered using a four-point Likert scale ranging from 0 (“Event never occurs”) to 3 (“Almost always”). Scale scores on the VDS ranged from 0 to 69. A higher score indicates a stronger visual discomfort.

The Glasgow Sensory Questionnaire (GSQ) [6], Japanese version [7], is a 42-item index of 14 subscales that assesses hypersensitivity and hyposensitivity in seven sensory modalities (Table 1). Initially, the GSQ was developed to investigate anomalous sensory processing in autism spectrum disorders, but it also can be applied to individuals with typical development [6], [7]. Each item is answered using a five-point Likert scale ranging from 0 (“Never”) to 4 (“Always”). The scores on each subscale ranged from 0 to 12. A higher score indicates a stronger hypersensitivity or hyposensitivity.

The Positive and Negative Affect Schedule (PANAS) [8], Japanese version [9], is a 20-item scale consisting of Positive Affect and Negative Affect subscales. By modifying the instructions [8], PANAS in this survey assessed the participants’ “general” emotional state (i.e., positive or negative mood on a daily basis). The original PANAS uses a five-point Likert scale (1 “Very slightly or not at all” to 5 “Extremely”), but the validated Japanese version uses a six-point scale ranging from 1 “Not applicable at all” to 6 “Very applicable”. Thus, in this survey, the scores on each subscale ranged from 10 to 60. A higher score indicates a more positive or negative trait mood.

The Disgust Propensity and Sensitivity Scale-Revised (DPSS-R) [10], Japanese version [11], is a 16-item index comprising Disgust Propensity and Disgust Sensitivity subscales (e.g., frequency with which the individual experiences disgust and the extent to which the individual feels uncomfortable because of disgust, respectively). Each item is answered using a five-point Likert scale ranging from 1 (“Never”) to 5 (“Always”). The subscale scores ranged from 8 to 40. A higher score indicates a stronger disgust propensity or sensitivity.
